# Using Caution When Interpreting Gender-Based Relative Risk. Comment on “The Effect of Cardiovascular Comorbidities on Women Compared to Men: Longitudinal Retrospective Analysis”

**DOI:** 10.2196/34647

**Published:** 2022-03-25

**Authors:** Imre Janszky

**Affiliations:** 1 Department of Public Health and Nursing Faculty of Medicine and Health Sciences Norwegian University of Science and Technology Trondheim Norway

**Keywords:** gender gap, sex differences, cardiovascular diseases, acute myocardial infarction, chronic ischemic heart disease, gender, diabetes, smoking, risk factors, comorbidities, relative risk, interaction

Dervic et al [1] have conducted an impressive, comprehensive analysis of Austrian health records to compare the relative risks of comorbidities for cardiovascular disease (CVD) among men and women. Based on the higher relative risks observed for most comorbidities among women, the authors suggested that “women appear to be more affected” by these comorbidities and that their findings may have public health relevance in designing screening and prevention strategies. However, theoretical epidemiological works have clearly shown that the heterogeneity of relative risks alone generally does not allow causal inference for interaction between exposures or evaluation of public health implications [2,3].

Consider the following intuitive example: in contrast to CVD or other diseases of complex multifactorial origin where the causes for individual cases are practically always obscure, let us consider a “disease” with a clear origin, namely homicide. In this example, we have an imaginary country with a northern and southern region. In both regions, there are 20 million inhabitants with an equal distribution of men and women (ie, 10 million men and 10 million women in each region). Normally, in both regions, 1 woman per million per year dies of homicide in this country; the corresponding number is 2 per million for men. However, last year, a serial killer started to operate exclusively in the northern region. He is a sniper shooting from far away; hence, he cannot see the sex of his victims. It follows that he does not discriminate between men and women. Last year, he killed 20 northerners: 10 men and 10 women. Thus, last year, the relative risk of being killed when we compare northerners to southerners will be 2 for women and 1.5 for men. Despite the higher relative risk for women, the sniper is clearly equally dangerous to both men and women.

When interpreting the findings of Dervic et al [1], we need to take into account the considerably higher baseline risk, that is, the excess risk of CVD without the respective comorbidities for the men in their study. Depending on the degree of male excess risk among the unexposed, the lower relative risk for CVD with comorbidities observed by the authors among men can also indicate no difference in the effect of these factors between the sexes or an even more detrimental effect in men compared with women.

## Abbreviations

CVD: cardiovascular disease

## References

[1] Dervic Elma, Deischinger Carola, Haug Nils, Leutner Michael, Kautzky-Willer Alexandra, Klimek Peter (2021). The Effect of Cardiovascular Comorbidities on Women Compared to Men: Longitudinal Retrospective Analysis. JMIR Cardio.

[2] Rothman KJ, Greenland S, Lash TL (2008). Modern Epidemiology.

[3] VanderWeele Tyler J, Robins James M (2007). The identification of synergism in the sufficient-component-cause framework. Epidemiology.

